# The benefits of systematic mapping to evidence-based environmental management

**DOI:** 10.1007/s13280-016-0773-x

**Published:** 2016-03-17

**Authors:** Neal R. Haddaway, Claes Bernes, Bengt-Gunnar Jonsson, Katarina Hedlund

**Affiliations:** 1 Mistra Council for Evidence-Based Environmental Management, Stockholm Environment Institute, Box 24218, 104 51 Stockholm, Sweden; 2 Department of Natural Sciences, Mid-Sweden University, 851 70 Sundsvall, Sweden; 3 Department of Biology, Lund University, 223 62 Lund, Sweden

**Keywords:** Evidence review, Secondary synthesis, Evidence-informed policy, Environmental policy, Forestry, Soil carbon

## Abstract

Reviews of evidence are a vital means of summarising growing bodies of research. Systematic reviews (SRs) aim to reduce bias and increase reliability when summarising high priority and controversial topics. Similar to SRs, systematic maps (SMs) were developed in social sciences to reliably catalogue evidence on a specific subject. Rather than providing answers to specific questions of impacts, SMs aim to produce searchable databases of studies, along with detailed descriptive information. These maps (consisting of a report, a database, and sometimes a geographical information system) can prove highly useful for research, policy and practice communities, by providing assessments of *knowledge gaps* (subjects requiring additional research), *knowledge gluts* (subjects where full SR is possible), and patterns across the research literature that promote best practice and direct research resources towards the highest quality research. Here, we introduce SMs in detail using three recent case studies that demonstrate their utility for research and decision-making.

**Electronic supplementary material:**

The online version of this article (doi:10.1007/s13280-016-0773-x) contains supplementary material, which is available to authorized users.

## Introduction

Synthesis of published research aims to build upon and go beyond primary studies to provide reliable answers to questions by formally summarising existing literature. Literature reviews are common in most research fields and, with an increasing use of statistical tools such as meta-analysis, these quantitative reviews provide a higher power for testing general predictions. Traditional literature reviews, including meta-analyses, however, are potentially susceptible to a range of possible biases, for example, selection bias, publication bias, and detection bias (Pullin and Stewart 2006).

**Fig. 1:**
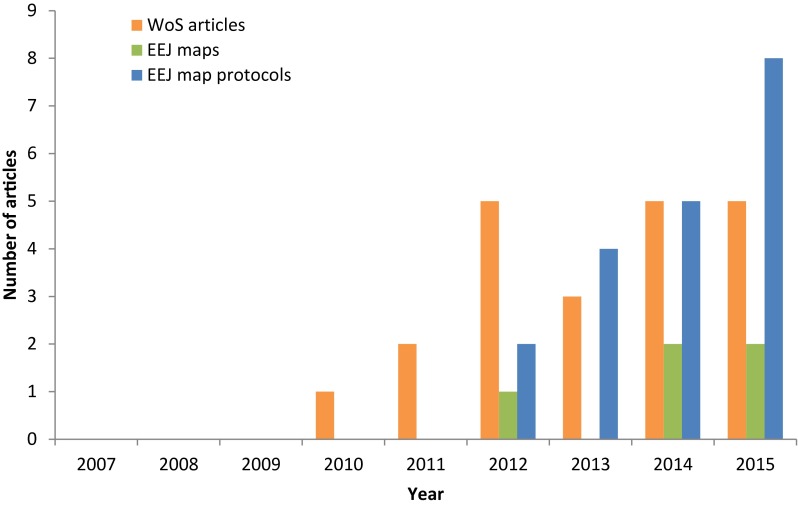
Published systematic map articles over time from Web of Science (WoS) (topic word search; “systematic map”) and systematic map protocols and reports published in *Environmental Evidence* (EEJ) (currently not indexed in WoS) (Haddaway et al. 2015a)

Systematic reviews (SRs) were established in the field of medicine in order to synthesise large bodies of primary research studies in a way that minimises bias, allowing for assessments of reliability, consensus and reasons for heterogeneity across the evidence base (The Cochrane Collaboration 2013). Since the establishment of the Cochrane Collaboration in 1992 (Allen and Richmond 2011), SR methods have become a ‘gold standard’ in evidence synthesis and SRs are now published at high rates (7 per day in the field of medicine; Bastian et al. 2010). Systematic review methods first applied to the field of conservation and environmental management in 2006 (Pullin and Stewart 2006), and since establishment of the Collaboration for Environmental Evidence (CEE) in 2008, the number of SRs published in environmental sciences has shown a steady increase (Fig. 1).

Whilst SRs are useful in providing estimates of effect sizes associated with specific interventions or exposures, systematic reviewing methods have also been adapted for use in answering questions relating to the state of the evidence base itself. Such questions aim to assess what research has been undertaken, which study settings have been examined and what methods have been used across an evidence base. These methods have been termed ‘systematic maps’ (SMs) (McKinnon et al. 2015). Systematic mapping was first undertaken in the social sciences (Bates et al. 2007; Clapton et al. 2009), but since the methods were adapted for use in environmental management and conservation (Randall and James 2012) they have become increasingly common. There are now 15 SM protocols published in the CEE journal *Environmental Evidence* (EEJ) (as of September 2015); an indication of the increasing attention paid to systematic mapping by commissioners and researchers alike. Here, we discuss the benefits of systematic mapping to research, policy and practice and provide two examples of recent SMs that demonstrate the high utility of the methods.

## The advent of systematic mapping in conservation and environmental management

Systematic maps were first developed and used to synthesise social science research (Bates et al. 2007; Clapton et al. 2009). Systematic mapping has now been adopted in a range of disciplines beyond the social sciences, and is the subject of considerable coordinated research effort within specific topics (e.g. spinal and brain injury research; Bragge et al. 2011). The first SM in environmental management research was published in 2012 (Randall and James 2012) and catalogued literature relating to the impacts of integrated farm management, organic farming and agri-environment schemes on biodiversity in temperate ecosystems. To date, 9 SMs have been published in EEJ (Randall and James 2012; Randall et al. 2015; Haddaway et al. 2014, 2015b; Roe et al. 2014; Bernes et al. 2015; Macura et al. 2015; Neaves et al. 2015). Interest in SM methods is expanding (Fig. 1): 15 SM protocols have been published since 2012 (as of September 2015; see Appendix S1), and a CEE Methods Group concerned with SMs has been recently formed (http://www.environmentalevidence.org/wp-content/uploads/2015/02/The-CEE-Systematic-Mapping-Methods-Group.docx).

## What is systematic mapping?

The objectives of SMs and SRs are fundamentally similar; to collate and describe all of the available published research evidence on a topic in an objective, repeatable and transparent manner (CEE 2013). These syntheses aim to be comprehensive and should be undertaken according to an a priori peer-reviewed method (a SM/SR protocol). Publication of a protocol that sets out the planned methodology before the review commences has a number of important benefits. Firstly, this essentially ‘registers’ the reviewers’ intent to complete a full review, which reduces chances of duplication of effort and can allow interested parties to contact the review team to provide advice, comments, evidence and critique for the review whilst it can still be considered and integrated into the review. Secondly, publication of protocols involves peer-review by subject and methodology experts, ensuring that the procedures to be used are as reliable as possible and that susceptibility to bias, such as publication bias and selection bias, are minimised. Finally, the publicly available protocol helps to ensure that the actual conduct of the review proceeds according to the protocol and does not deviate from the initial plans.

Generally, SMs are appropriate for broad topics that are often too expansive for an individual SR, and typically answer questions such as: “what evidence exists concerning…?”, “how much research is available regarding…?”, and “what is the current state of knowledge about…?”. Systematic maps do not aim to provide a quantitative or qualitative answer to a question of impacts (i.e. a summary effect estimate) or test a hypothesis, but rather an overview of the evidence base, or more specifically, what research has been undertaken, where and how.

Procedurally, SMs and SRs share many similarities (Table 1). The process of question formulation, protocol development, searching and screening stages are essentially the same. However, while extraction of descriptive data (also known as meta-data) is an integral step in SM, extraction of study findings (quantitative or qualitative results) is typically not performed. Consequently, synthesis in SMs is limited to a narrative description of the state of the evidence base, with no quantitative or qualitative analysis of study findings. Critical appraisal (the formalised assessment of reliability and risk of bias in individual studies) may be performed to some extent, but this is typically restricted to an assessment of study internal validity (quality or susceptibility to bias), since external validity (generalisability) cannot typically be assessed for the broad topics often investigated with SMs.

**Table 1:** Key differences between systematic maps and systematic reviews according to the procedural steps used. ‘Key elements’ refer to the population, intervention or exposure, comparator and outcome components of the study question

	Systematic reviews	Systematic maps
Objective	Question concerns the efficacy of an environmental management intervention or impact of an exposure	Question concerns the state of the evidence base for a specific topic (commonly based on one or more related interventions or exposures)
The topic	Typically narrow, focused question with single/few interventions/exposures and single/few outcomes	Typically broader question involving multiple interventions/exposures and/or multiple outcomes
Searches for evidence	Search terms specified for most key elements, resulting in a moderate volume of evidence	More sensitive (wider reaching) search string with some key element terms not strictly specified, resulting in a larger volume of evidence
Study inclusion	Inclusion criteria typically specified in detail and defined for all key elements	Inclusion criteria may not be explicitly defined for all key elements, possibly being included iteratively during the review
Data extraction	Complete extraction of meta-data and study findings (qualitative or quantitative)	Extraction of meta-data only
Critical appraisal	Assessment of internal validity (quality) and external validity (generalisability) performed for all included studies	Study internal validity may be appraised but generalisability typically not assessed
Synthesis	Narrative synthesis of the evidence base along with quantitative or qualitative synthesis of study findings	Narrative synthesis of the evidence base but no synthesis of study findings
Key review outputs	Qualitative and quantitative (where possible) summary effect estimated, implications for policy/practice, implications for research	Searchable database of relevant studies, implications for research (primary/secondary), and making the knowledge base available to policy/practice

## Applicability of systematic mapping to environmental management

Translating systematic mapping and associated SR methodology from the social sciences and medicine, respectively, to environmental sciences requires some careful consideration of the relevant analogous systems that we deal with. However, these considerations are not as challenging as often perceived. For example, the PICO/PECO (population, intervention/exposure, comparator, outcome) model used in SRs and SMs translates well onto environmental subjects: population refers to the specific system investigated, rather than a human population (e.g. farms in boreo-temperate systems); intervention/exposure refers to either a management practice or some other environmental factor (e.g. soil tillage using mouldboard ploughing); comparator refers to the factor with which an intervention/exposure is compared (e.g. before tillage or untilled control group), although SMs may not always require the presence of a comparator; outcome refers to the variable being measured (e.g. soil carbon concentration). Not all review questions have a PICO/PECO structure, of course, but this is not particular to environmental science. There is a mistaken belief that SR methods are only appropriate for randomised control trials (RCTs) and quantitative, experimental research. In fact, SRs and SMs can be used to synthesise any form of research evidence, including observation and qualitative studies (Haddaway and Bilotta 2015).

## Systematic map outputs and uses

Whilst key SR outputs include quantitative or qualitative summary effect estimates for the interventions or exposures of interest, the main output from a SM is the description of the evidence base. This description should take the form of the SM report and a searchable database of relevant studies. The report may include a discussion of the following key aspects of the evidence base: (i) general patterns in study methods and settings, (ii) knowledge gluts, where substantial numbers of studies have investigated a similar subtopic, (iii) knowledge gaps, where there is a significant lack of research on a subtopic, and (iv) deficiencies or best practices in research methodology (although this latter point is typically the result of critical appraisal, which is an optional stage in SMs). The SM database includes a range of descriptive information for all included studies, including: (i) citation information, (ii) study setting descriptors, (iii) methodology details, and (iv) summaries of the nature and location of quantitative or qualitative study findings (but, importantly, not the findings themselves). The SM database is intended to be a readily usable resource for researchers and decision-makers when looking for evidence, and usability is a main consideration when compiling the SM and building the database.

In addition to the report and map database, reviewers may also produce other outputs from the systematic mapping process. In particular, geographical information systems (GISs) allow for information from within the SM database to be displayed across a cartographic map. This approach can be particularly useful for environmental topics that are global or wide scale in nature.

Systematic maps have a variety of uses across research, policy and practice. Researchers, research funders and decision-makers can benefit from learning about knowledge gaps, subtopics that are underrepresented in the evidence base. Such gaps may warrant novel primary research in order to provide a future evidence base, particularly where prevailing policy or practice is controversial or perceived to require change. Researchers, research funders and decision-makers can also benefit from highlighted knowledge gluts that allow full synthesis in the form of SR. Furthermore, areas that have received substantial research effort may be deemed by research funders to be sufficiently well understood that research funding could be more effectively directed elsewhere. Researchers and research funders can also benefit from the identification of deficiencies and best practices across the evidence base, which may be used to increase consistency across studies. Environmental managers may also benefit from using the SM database as a library, from which they can identify a subset of studies that are most relevant to their situation. Finally, since SMs published with CEE are Gold Open Access (i.e. freely, immediately accessible in full to everyone), researchers, practitioners and policy-makers can use the database as a source of detailed descriptive information regarding studies that may not be individually accessible in full text. Practitioners and policy-makers often cite limited accessibility as a barrier to evidence use (e.g. Haynes and Haines 1998; Oliver et al. 2014), and the provision of accessible, reliable summaries may prove particularly important for environmental decision-makers.

Besides the CEE journal (EEJ), other options exist for publication of SMs. However, since SRs and SMs are new methods, publishing them without using the expertise of a coordinating body (such as the CEE and its associated journal EEJ) can have serious disadvantages. Firstly, no other formal academic publication as yet accepts SM or SR protocols. Secondly, other journals may not have the methodological expertise necessary to identify flaws or missing information within review reports. This is evident in a recent example, a SM on the subject of European agroforestry ecosystem services that was published in the journal Ecological Indicators (Fagerholm et al. 2016). Whilst this SM purports to be undertaken according to CEE guidelines, it lacks a peer-reviewed protocol. In this case, the authors have chosen not to include grey literature, and their search strategy involves the use of country names within the search string. The former practice opens the review to publication bias [the inclusion of only academic research is well-proven to be more likely to show positive and significant findings (Rothstein et al. 2006)]. The latter practice compromises comprehensiveness, since there is a significant risk of missing research that does not mention the study country within its title, abstract or keywords. It is possible to self-publish a SM protocol, however, particularly where independent peer-review by subject and methodology experts can be transparently demonstrated. Some generalist journals are also increasingly able to peer-review and publish SRs and SMs, such as PLoS One, which requires review authors to submit a PRISMA checklist (a set list of descriptors that confirm the review has undertaken specific required aspects of formal review methodology). Such checklists are not a definitive indication of reliability on their own, however (O’Leary et al. 2015).

Review questions initiated by external commissioners (e.g. Defra; Randall et al. 2015) are often very broad in nature and may initially be suitable for review by SM. Furthermore, since SMs can highlight knowledge gaps and knowledge gluts, they may be a useful first step before full SR where the volume of evidence on a topic is poorly understood. Completing a full SR following on from a SM may then be a much more rapid process than for a totally novel topic, since many of the SR processes have already been undertaken. Furthermore, SMs may provide a resource from which a number of full SR topics can be identified. Although a useful first step, SMs are not necessarily any less resource intensive than SRs. Despite not involving full data extraction, critical appraisal or quantitative/qualitative synthesis, SMs typically cover broader evidence bases and may assimilate a larger number of studies.

SMs also prove useful additions to full SR. Tables of relevant studies and their descriptive meta-data are likely to be produced by the vast majority of reviewers as part of the SR process. Providing these resources as supplementary, interactive, searchable databases would be a valuable output for all reviews, requiring minimal additional effort. Furthermore, reviewers may choose to produce a map database that is broader in scope than the subset of studies taken on to full synthesis, increasing the relevance to end-users.

In summary, SMs have a plethora of uses to researchers, research funders, policy-makers and practitioners alike: from acting as a library for finding single relevant studies to providing recommendations of knowledge gaps that may warrant further research.

## Systematic map case studies

Three new SMs have recently been completed on the subjects of agricultural impacts on soil organic carbon (SOC) (Haddaway et al. 2015b), management of protected forests (Bernes et al. 2015) and on-farm mitigation measures for improving water quality (Randall et al. 2015). These maps have used state-of-the-art methodology in systematic mapping, including in-depth stakeholder engagement from the outset of the review projects, comprehensive assessment of all relevant review bibliographies, the use of GIS to visually display the contents of the map databases. These projects demonstrate the utility of SMs.

### Impacts of agricultural management on soil organic carbon (SOC)

Swedish stakeholders, including the Swedish Board of Agriculture and the Swedish Environmental Protection Agency, identified the need to better understand the relationship between management practices on arable farmland and stocks of SOC. A SR was initiated, focusing on research from the warm temperate and snow climate zones (according to the Köppen–Geiger climate classification; Kottek et al. 2006), and based on a predetermined methodology set out in a detailed protocol published in EEJ (Söderström et al. 2014). When the search strategy was implemented, however, the volume of evidence returned was extensive, and a decision was made for practical reasons of resource availability to produce a SM first. This map described studies across a range of agricultural management practices (soil amendments, crop rotation, fertiliser application and tillage), with interventions identified iteratively where study length was 10 years or more (to ensure SOC changes were given time to manifest themselves).

A total of 740 articles were included in the SM (24547 search results > 5735 relevant titles > 1814 relevant abstracts > 740 relevant full texts). One of the outputs of this SM was a map report, which described the background, methods and results of the mapping exercise, and discussed the range and nature of the evidence base. In addition, a SM database was published, providing details relating to the citation of the article describing the study, the study setting, the experiment studied, the methodology used to measure the experiment soil conditions, and the location, units and format of the quantitative findings of the study. Furthermore, a web-based GIS was produced based on the contents of the map. This GIS allows users to filter subsets of studies on a spatial map (as opposed to a metaphorical evidence map), forming a different and user-friendly interface to the database.

The SM authors highlighted several knowledge gaps (e.g. a paucity of studies from Russia) and knowledge gluts (e.g. a multitude of studies investigating conservation tillage), and noted a lack of spatial and temporal replication and frequently missing information (such as study methods, location and description of the interventions) within included articles. This information was detailed within the SM report and may prove useful for primary researchers (knowledge gaps, methodological deficiencies), secondary researchers (knowledge gluts), research funders (knowledge gluts, knowledge gaps, research deficiencies) and decision-makers (knowledge gaps, knowledge gluts) alike. Following on from the SM, the research team behind the review is currently undertaking two full SRs on subsets of the evidence identified in the map. One review will synthesise the findings of studies investigating the relative impacts of different tillage intensities on soil organic carbon. The second review will include all interventions, but will focus purely on studies with long-time series data (i.e. 30 years or more, with multiple measurements through time). These two full reviews will involve an update to the original searches to ensure that recently published evidence is included. Both reviews will also include a full quantitative synthesis (i.e. meta-analysis). Further knowledge gluts were identified in the SM that may also be synthesised by the review team if resources allow.

### Impacts of active management on biodiversity in forests set aside for conservation or restoration

Conservationists in Sweden and several other countries are currently involved in a discussion of the best means of preserving or restoring forest biodiversity in reserves and other areas that have been set aside from commercial forestry. One management option is non-intervention; other options include various forms of active management such as prescribed burning, thinning, partial harvesting, grazing or exclusion from grazing. Current practices and recommendations for the management of forest set-asides are often based on traditions (i.e. the “free-development” paradigm) rather than scientific evidence, however. Swedish stakeholders (including County administrators, landowners and environmental NGOs) therefore suggested a SR of all available evidence on the biodiversity effects of relevant forms of management in cool temperate and boreal forests.

Since the evidence base was likely to be quite heterogeneous, including studies of a variety of interventions and many different aspects of biodiversity, it was recognised from the outset that systematic mapping might be useful as a first step towards full SR of specific management options. The review team searched not only for studies of interventions in actual forest set-asides, but also for appropriate evidence from commercially managed forests, since some practices applied there may equally be useful for conservation or restoration purposes.

Around 800 relevant studies were found (16 484 search results > 6142 relevant titles > 1762 relevant abstracts > 798 relevant full texts), almost two-thirds of which had been conducted in North America. Most of the rest had been performed in Central or Northern Europe. These studies were presented much in the same way as those in the SOC SM described above, i.e. in a SM report, an associated database with details of each study, and a separate GIS which made it possible to plot and identify all included studies (or any selection of them) on a cartographic map. The details provided about the studies included descriptive data (meta-data) on locations, study design, forest stands, interventions, types of biodiversity outcomes and focal species.

Knowledge gaps identified within the SM included a lack of studies on hydrological interventions (such as restoration of forested wetlands) and traditional silvicultural systems that are presently uncommon (such as coppicing and pollarding). As in the SOC SM, there was also a paucity of useful Russian studies.

Based on the availability of relevant studies, the existence or absence of earlier reviews, and needs expressed by stakeholders, the authors of the SM finally identified four subtopics for which it would be feasible to complete full SRs: (1) What are the impacts of thinning, partial harvesting and understorey removal on the diversity of ground vegetation in mature temperate and boreal forest? (2) What are the impacts of temperate and boreal forest stand- and tree-scale interventions on dead wood and saproxylic species? (3) What is the effect of prescribed burning in temperate and boreal forest on biodiversity, beyond tree regeneration, pyrophilous and saproxylic species? and (4) What are the impacts of manipulating the pressure of grazing and browsing by livestock or wild ungulates on the diversity of temperate and boreal forest plants and invertebrates?

### Effectiveness of on-farm mitigation measures for improving water quality

Agriculture contributes high amounts of nitrogen, phosphorous, sediments, pesticides, and with livestock also potential human pathogens to waterways that all contribute to a decline in water quality (Edwards and Withers 2008; Kay et al. 2008; Collins et al. 2009; Defra 2009). This decline can directly impact the environment and its associated ecosystem services, whilst also taxing limited government funding available for environmental management. EU member states are obliged under the Water Framework Directive (WFD) to mitigate water pollution, but as yet no systematic approach has been made to identify and assess the various mitigation measures used.

In their SM, Randall et al. (2015) collate research from temperate countries pertaining to six key mitigation measures: (1) slurry storage, (2) catch crops, (3) woodland creation, (4) controlled trafficking, (5) subsoiling and (6) vegetated buffer strips.

The reviewers included 718 studies in the map (74 086 search records > 1359 relevant titles > 718 relevant abstracts > 495 relevant full texts). Buffer strips were the most frequently studied of the six interventions, with cover crops and slurry storage also commonly investigated. Very few/no studies had focused on woodland creation, controlled trafficking and subsoiling. In terms of measured outcomes, nitrogen was most frequently studied, followed by phosphorus, sediment, pesticides and bacterial pathogens.

The majority of research in this area was found to focus on mitigating nitrogen pollution and on the use of buffer strips and catch crops. Knowledge gaps were therefore found for the remaining four mitigation interventions and outcomes other than nitrogen. Furthermore, the reviewers found relatively few robust studies: few used long-term datasets, measured across seasons, possessed well-matched controls, measured baseline data, or sampled within fields and watercourses. These research quality gaps identify the benefit that would come from adding more reliable research to the evidence base.

## Conclusions

SMs are becoming increasingly popular in environmental sciences and are likely to be as influential and prevalent as SRs. The outputs of SMs are useful for a range of stakeholders, but most importantly those that might benefit from highlighted knowledge gaps, knowledge gluts and suggested improvements and best practices in research methods. Thus, SMs are not only useful to researchers (primary and secondary) but also funders, policy-makers and practitioners. SRs will almost certainly always be more widely applicable in practice by stakeholders, since they aim to provide summary effect estimates of influential interventions or exposures. However, a strong need for systematic mapping remains, not least as a useful first step in the SR process, particularly where broad concerns are highlighted by commissioners and stakeholders.

## Electronic supplementary material

Below is the link to the electronic supplementary material.

Supplementary material 1 (PDF 428 kb)

## References

[CR1] Allen C, Richmond K (2011). The Cochrane Collaboration: International activity within Cochrane Review Groups in the first decade of the twenty-first century. Journal of Evidence-Based Medicine.

[CR2] Bastian H, Glasziou P, Chalmers I (2010). Seventy-five trials and eleven systematic reviews a day: how will we ever keep up?. PLoS Medicine.

[CR3] Bates S, Clapton J, Coren E (2007). Systematic maps to support the evidence base in social care. Evidence and Policy.

[CR4] Bernes CGB, Jonsson K, Junninen A, Lõhmus E, Macdonald J Müller, Sandström J (2015). What is the impact of active management on biodiversity in forests set aside for conservation or restoration? A systematic map. Environmental Evidence.

[CR5] Bragge P, Clavisi O, Turner T, Tavender E, Collie A, Gruen RL (2011). The global evidence mapping initiative: scoping research in broad topic areas. BMC Medical Research Methodology.

[CR6] Clapton J., D. Rutter, and N. Sharif. 2009. SCIE Systematic mapping guidance; April 2009. www.scie.org.uk/publications/researchresources/rr03.pdf.

[CR8] Collaboration for Environmental Evidence. 2013. Guidelines for Systematic Review and Evidence Synthesis in Environmental Management. Version 4.2. Environmental Evidence: www.environmentalevidence.org/Documents/Guidelines/Guidelines4.2.pdf.

[CR9] Collins AL, Anthony SG, Hawley J, Turner T (2009). Predicting potential change in agricultural sediment inputs to rivers across England and Wales by 2015. Marine & Freshwater Research.

[CR10] Defra. 2009. Safeguarding our soils. Department of Environment, Food and Rural Affairs (Defra), UK.

[CR11] Edwards A, Withers P (2008). Transport and delivery of suspended solids, nitrogen and phosphorus from various sources to freshwaters in the UK. Journal of Hydrology.

[CR12] Fagerholm N, Torralba M, Burgess PJ, Plieninger T (2016). A systematic map of ecosystem services assessments around European agroforestry. Ecological Indicators.

[CR13] Haddaway NR, Bilotta GS (2015). Systematic reviews: Separating fact from fiction. Environment International.

[CR14] Haddaway NR, Styles D, Pullin AS (2014). Evidence on the environmental impacts of farm land abandonment in high altitude/mountain regions: a systematic map. Environmental Evidence.

[CR16] Haddaway NR, Hedlund K, Jackson LE, Kätterer T, Lugato E, Thomsen IK, Jørgensen HB, Söderström B (2015). What are the effects of agricultural management on soil organic carbon in boreo-temperate systems?. Environmental Evidence.

[CR15] Haddaway NR, Woodcock P, Macura B, Collins A (2015). Making literature reviews more reliable through application of lessons from systematic reviews. Conservation Biology.

[CR17] Haynes B, Haines A (1998). Getting research findings into practice: Barriers and bridges to evidence based clinical practice. British Medical Journal.

[CR18] Kay D, Crowther J, Fewtrell L, Francis C, Hopkins M, Kay C, McDonald AT, Stapleton CM, Watkins J, Wilkinson J, Wyer MD (2008). Quantification and control of microbial pollution from agriculture: a new policy challenge?. Environmental Science & Policy.

[CR19] Kottek M, Grieser J, Beck C, Rudolf B, Rubel F (2006). World map of the Koppen–Geiger climate classification updated. Meteorologische Zeitschrift.

[CR20] Macura B, Secco L, Pullin AS (2015). What evidence exists on the impact of governance type on the conservation effectiveness of forest protected areas? Knowledge base and evidence gaps. Environmental Evidence.

[CR21] McKinnon MC, Cheng SH, Garside R, Masuda YJ, Miller DC (2015). Sustainability: Map the evidence. Nature.

[CR22] Neaves LE, Eales J, Whitlock R, Hollingsworth PM, Burke T, Pullin AS (2015). The fitness consequences of inbreeding in natural populations and their implications for species conservation–a systematic map. Environmental Evidence.

[CR23] O’Leary BC, Bayliss HR, Haddaway NR (2015). Beyond PRISMA: Systematic reviews to inform marine science and policy. Marine Policy.

[CR24] Oliver K, Innvar S, Lorenc T, Woodman J, Thomas J (2014). A systematic review of barriers to and facilitators of the use of evidence by policymakers. BMC Health Services Research.

[CR25] Pullin AS, Stewart GB (2006). Guidelines for systematic review in conservation and environmental management. Conservation Biology.

[CR26] Randall NP, James KL (2012). The effectiveness of integrated farm management, organic farming and agri-environment schemes for conserving biodiversity in temperate Europe—A systematic map. Environmental Evidence.

[CR27] Randall NP, Donnison LM, Lewis PJ, James KL (2015). How effective are on-farm mitigation measures for delivering an improved water environment? A systematic map. Environmental Evidence.

[CR28] Roe D, Fancourt M, Sandbrook C, Sibanda M, Giuliani A, Gordon-Maclean A (2014). Which components or attributes of biodiversity influence which dimensions of poverty. Environmental Evidence.

[CR29] Rothstein HR, Sutton AJ, Borenstein M (2006). Publication bias in meta-analysis: Prevention, assessment and adjustments.

[CR30] Söderström B, Hedlund K, Jackson LE, Kätterer T, Lugato E, Thomsen IK, Jørgensen HB (2014). What are the effects of agricultural management on soil organic carbon (SOC) stocks?. Environmental Evidence.

[CR7] The Cochrane Collaboration. 2013. History. Accessed September 2014, from http://www.cochrane.org/about-us/history.

