# Supplementary material for: The benefits of systematic mapping to evidence-based environmental management
Source: Ambio. 2016 Mar 17;45(5):613–20. doi: 10.1007/s13280-016-0773-x (PMC4980318; doi:10.1007/s13280-016-0773-x)
Supplement: Supplementary file 1 — Supplementary material 1 (PDF 428 kb) [file 13280_2016_773_MOESM1_ESM.pdf]

Ambio

Electronic Supplementary Information
This supplementary information has not been peer-reviewed

Title: **Appendix S1. List of systematic map protocols published by CEE in the journal Environmental Evidence (as of December 2015)**

1. Doody, D.G., Augustenborg, C.A., Withers, P.J. and Crosse, S., 2015. A systematic map protocol: What evidence exists to link agricultural practices with ecological impacts for Irish waterbodies?. Environmental Evidence, 4(1), p.1.
2. Nguyen, H., Herbohn, J., Clendenning, J., Lamb, D., Dressler, W., Vanclay, J. and Firn, J., 2015. What is the available evidence concerning relative performance of different designs of mixed-species plantings for smallholder and community forestry in the tropics? A systematic map protocol. Environmental Evidence, 4(1), p.1.
3. Leisher, C., Temsah, G., Booker, F., Day, M., Agarwal, B., Matthews, E., Roe, D., Russell, D., Samberg, L., Sunderland, T. and Wilkie, D., 2015. Does the gender composition of forest and fishery management groups affect resource governance and conservation outcomes: a systematic map protocol. Environmental Evidence, 4(1), p.1.
4. Cerutti, P.O., Sola, P., Chenevoy, A., Iiyama, M., Yila, J., Zhou, W., Djoudi, H., Atyi, R.E., Gautier, D.J., Gumbo, D. and Kuehl, Y., 2015. The socioeconomic and environmental impacts of wood energy value chains in Sub-Saharan Africa: a systematic map protocol. Environmental Evidence, 4(1), p.12.
5. Thorn, J., Snaddon, J., Waldron, A., Kok, K., Zhou, W., Bhagwat, S., Willis, K. and Petrokofsky, G., 2015. How effective are on-farm conservation land management strategies for preserving ecosystem services in developing countries? A systematic map protocol. Environmental Evidence, 4(1), pp.1-13.
6. Bayliss, H.R., Schindler, S., Essl, F., Rabitsch, W. and Pullin, A.S., 2015. What evidence exists for changes in the occurrence, frequency or severity of human health impacts resulting from exposure to alien invasive species in Europe? A systematic map protocol. Environmental Evidence, 4(1), p.10.
7. Schoneveld, G., Di Matteo, F., Brandao, F., Pacheco, P., Jelsma, I. and Jarnholt, E.D., 2015. A systematic mapping protocol: what are the impacts of different upstream business models in the agriculture and forestry sector on sustainable development in tropical developing countries?. Environmental Evidence, 4(1), p.1.
8. Reed, J., Deakin, L. and Sunderland, T., 2015. What are 'Integrated Landscape Approaches' and how effectively have they been implemented in the tropics: a systematic map protocol. Environmental Evidence, 4(2).
9. Garcia-Yi, J., Lapikanonth, T., Vionita, H., Vu, H., Yang, S., Zhong, Y., Li, Y., Nagelschneider, V., Schlindwein, B. and Wesseler, J., 2014. What are the socio-economic impacts of genetically modified crops worldwide? A systematic map protocol. Environmental Evidence, 3(1), p.24.
10. Bottrill, M., Cheng, S., Garside, R., Wongbusarakum, S., Roe, D., Holland, M., Edmond, J. and Turner, W.R., 2014. What are the impacts of nature conservation interventions on human well-being: a systematic map protocol. Environ Evid, 3, p.16.

11. Gathmann, A. and Priesnitz, K.U., 2014. What is the evidence on the inheritance of resistance alleles in populations of lepidopteran/coleopteran maize pest species: a systematic map protocol. Environmental Evidence, 3(1), p.13.
12. Haddaway, N.R., Styles, D. and Pullin, A.S., 2013. Environmental impacts of farm land abandonment in high altitude/mountain regions: a systematic map of the evidence. Environ Evid, 2(18), pp.1-18.
13. Roe, D., Sandbrook, C., Fancourt, M., Schulte, B., Munroe, R. and Sibanda, M., 2013. A systematic map protocol: which components or attributes of biodiversity affect which dimensions of poverty. Environmental Evidence, 2(8).
14. Roberts, P.D., Diaz-Soltero, H., Hemming, D.J., Parr, M.J., Wakefield, N.H. and Wright, H.J., 2013. What is the evidence that invasive species are a significant contributor to the decline or loss of threatened species? A systematic review map. Environmental Evidence, 2(5), p.7.
15. Munroe, R., Roe, D., Doswald, N., Spencer, T., Möller, I., Vira, B., Reid, H., Kontoleon, A., Giuliani, A., Castelli, I. and Stephens, J., 2012. Review of the evidence base for ecosystem-based approaches for adaptation to climate change. Environmental Evidence, 1(1), p.13.
